# Substitution of Human Papillomavirus Type 16 L2 Neutralizing Epitopes Into L1 Surface Loops: The Effect on Virus-Like Particle Assembly and Immunogenicity

**DOI:** 10.3389/fpls.2019.00779

**Published:** 2019-06-20

**Authors:** Aleyo Chabeda, Albertha R. van Zyl, Edward P. Rybicki, Inga I. Hitzeroth

**Affiliations:** ^1^ Biopharming Research Unit, Department of Molecular and Cell Biology, University of Cape Town, Cape Town, South Africa; ^2^ Institute of Infectious Disease and Molecular Medicine, University of Cape Town, Cape Town, South Africa

**Keywords:** HPV-16, L1:L2 chimera, L2 substitution, epitope display, plant-produced, cross-neutralization

## Abstract

Cervical cancer caused by infection with human papillomaviruses (HPVs) is the fourth most common cancer in women globally, with the burden mainly in developing countries due to limited healthcare resources. Current vaccines based on virus-like particles (VLPs) assembled from recombinant expression of the immunodominant L1 protein are highly effective in the prevention of cervical infection; however, these vaccines are expensive and type-specific. Therefore, there is a need for more broadly protective and affordable vaccines. The HPV-16 L2 peptide sequences 108-120, 65-81, 56-81, and 17-36 are highly conserved across several HPV types and have been shown to elicit cross-neutralizing antibodies. To increase L2 immunogenicity, L1:L2 chimeric VLPs (cVLP) vaccine candidates were developed. The four L2 peptides mentioned above were substituted into the DE loop of HPV-16 L1 at position 131 (SAC) or in the C-terminal region at position 431 (SAE) to generate HPV-16-derived L1:L2 chimeras. All eight chimeras were transiently expressed in *Nicotiana benthamiana via Agrobacterium tumefaciens*-mediated DNA transfer. SAC chimeras predominantly assembled into higher order structures (*T* = 1 and *T* = 7 VLPs), whereas SAE chimeras assembled into capsomeres or formed aggregates. Four SAC and one SAE chimeras were used in vaccination studies in mice, and their ability to generate cross-neutralizing antibodies was analyzed in HPV pseudovirion-based neutralization assays. Of the seven heterologous HPVs tested, cross-neutralization with antisera specific to chimeras was observed for HPV-11 (SAE 65-18), HPV-18 (SAC 108-120, SAC 65-81, SAC 56-81, SAE 65-81), and HPV-58 (SAC 108-120). Interestingly, only anti-SAE 65-81 antiserum showed neutralization of homologous HPV-16, suggesting that the position of the L2 epitope display is critical for maintaining L1-specific neutralizing epitopes.

## Introduction

Approximately one in six global deaths is due to cancer, with the economic cost estimated at US$1.2 trillion in 2010 (World Health Organisation, 2017). Cancer is the second leading cause of death (Abubakar et al., 2015) and it was estimated that human papillomavirus (HPV)-related cancers account for 5% of all human cancers (De Martel et al., 2012). Cervical cancer is the fourth most common cancer in women globally and results in an estimated 567,000 cases and 311,000 deaths every year (Bray et al., 2018). About 80% of these cases occur in developing countries, largely due to limited healthcare resources. Most HPV infections are cleared by the immune system (Goodman et al., 2008; Rosa et al., 2008); however, some benign cervical lesions progress to invasive cervical cancer (ICC) caused predominantly by high-risk HPVs (zur Hausen, 2002). High-risk HPV-16 and HPV-18 are the most common cause of ICC and are associated with 70% of cervical cancer cases (Smith et al., 2007; de Sanjose et al., 2010), but at least 13 other high-risk HPVs cause cancer (zur Hausen, 2002; Parkin and Bray, 2006).

HPVs are small non-enveloped double-stranded DNA viruses with a genome size of approximately 8 kb (de Villiers et al., 2004) and infect mucosal and cutaneous basal epithelial cells after tissue microtrauma (Kines et al., 2009). The capsid is arranged in a *T* = 7 icosahedral formation and consists of major and minor capsid proteins, L1 and L2, respectively (Conway and Meyers, 2009). The major capsid protein consists of 360 copies of L1 that assembles into 72 pentamers and up to 72 copies of L2 can be integrated into each capsid (Buck et al., 2005, 2008). L1 assembles into virus-like particles (VLPs) in the presence or absence of the L2 minor capsid protein. VLPs retain the immunological properties of native papillomaviruses (Kirnbauer et al., 1992; Hagensee et al., 1993; Casini et al., 2004) and produce high titers of neutralizing antibodies (nAbs) when used as a vaccine (Christensen et al., 1994; Roden et al., 2000).

Three prophylactic vaccines: Cervarix™, a bivalent HPV-16/18 VLP vaccine; Gardasil^®^, a quadrivalent HPV-6/11/16/18 VLP vaccine; and Gardasil^®^9, a nonavalent HPV-6/11/16/18/31/33/45/52/58 VLP vaccine, based on the immunodominant L1 major capsid protein are currently on the market and have been shown to be effective in preventing cervical disease (Naud et al., 2014; Huh et al., 2017); however, the global burden of cervical cancer remains high, particularly in low-resource countries due to vaccine cost, type specificity of the vaccines, and poor screening and treatment programs. Although the most recent Gardasil^®^9 vaccine should address the low cross-neutralization observed with original vaccines, the addition of more L1 VLP types has not decreased the cost of current vaccines. Hence, there is a need for next-generation HPV vaccines that broadly target oncogenic HPV types, at reduced cost to women particularly in developing countries suffering most from cervical cancer (Roden and Stern, 2018) and penile cancer in men (Cardona and García-Perdomo, 2018).

Next-generation vaccines using L2 peptides have been investigated to generate more cross-protective responses (Schellenbacher et al., 2017). Anti-L2 antibodies can neutralize a broad range of mucosal and cutaneous HPVs (Pastrana et al., 2005; Alphs et al., 2008), suggesting that a L2 vaccine could address the type-restrictive efficacy of L1 vaccines. The N-terminus of HPV-16 L2 has a highly conserved region from amino acids (aa) 1-120 (Lowe et al., 2008), and L2 peptides 108-120 (Kawana et al., 1999), 65-81 (Jagu et al., 2013), 56-81 (Kawana et al., 1998; Kondo et al., 2007, 2008; Slupetzky et al., 2007), and 17-36 (Gambhira et al., 2007; Kondo et al., 2007, 2008; Alphs et al., 2008; Schellenbacher et al., 2009) have been shown to elicit nAbs that cross-neutralize other HPV types and provide protection against passive challenge. However, L2 is immunologically subdominant to L1, therefore scaffolded display of L2 peptides and the construction of chimeric proteins with L1 has been used to overcome these limitations. The structure and assembly of L1 has been well described (Chen et al., 2000b; Modis et al., 2002; Bishop et al., 2007) and L1 surface-exposed regions contain the conformational epitopes involved in the production of nAbs (Christensen et al., 1994, 1996; Roden et al., 1997; White et al., 1999). Several studies have shown that the insertion or substitution of several peptides into several L1 surface loops does not affect chimeric VLP (cVLP) assembly, with both anti-L1 and anti-L2 responses observed (Slupetzky et al., 2001, 2007; Sadeyen et al., 2003; Varsani et al., 2003; Schellenbacher et al., 2009, 2013; McGrath et al., 2013; Pineo et al., 2013; Chen et al., 2018). The insertion of the HPV-16 L2 peptide aa 17-36 (RG-1) in the L1 DE surface loop has shown the most promise as a candidate cVLP vaccine as it has been shown to protect mice against challenge with high-risk mucosal pseudovirion (PsV) types HPV-16/18/45/31/33/52/58/35/39/51/59/68/56/73/26/53/66/34 and low-risk types HPV-6/43/44, with protection observed 1 year after vaccination (Schellenbacher et al., 2013). This candidate vaccine is currently under cGMP production and is expected to enter a phase I clinical trial soon (Buchman et al., 2016; Roden and Stern, 2018).

Plants provide a convenient protein production platform to potentially reduce the cost of vaccine production compared to traditional microbial fermentation or mammalian/insect cell expression systems. Their production is easily scalable, they are eukaryotes that contain the necessary machinery for mammal-like post-translational modification, and they have no risk of contamination by human pathogens (Biemelt et al., 2003; Fischer et al., 2004; Rybicki, 2010). HPV VLPs have been successfully produced in plants *via* transient expression (Varsani et al., 2006b; Maclean et al., 2007; Regnard et al., 2010; Matic et al., 2011; Pineo et al., 2013), and have been shown to be immunogenic and protective in animal models (Kohl et al., 2006). Furthermore, L1:L2 cVLPs (L2 substituted in the h4 helix of L1) previously produced in our group by Pineo et al. (2013) were shown to assemble into higher order structures, and elicit anti-L1 and anti-L2 antibody responses which neutralized HPV-16 and HPV-52 PsVs.

In this study, we report the purification of five plant-produced HPV-16 L1:L2 cVLPs with L2 substituted in the DE loop (SAC) or the C-terminal region of L1 between the h4 and β-J structural region (SAE), based on insect-cell produced chimeras described by Varsani et al. (2003). The effect of L2 peptide substitution on chimera assembly and presentation of L1 epitopes was analyzed, and the immunogenicity and the cross-neutralizing potential of the cVLPs investigated.

## Materials and Methods

### Large-Scale Expression of L1:L2 Chimeras in *Nicotiana benthamiana*

**Figure 1:**
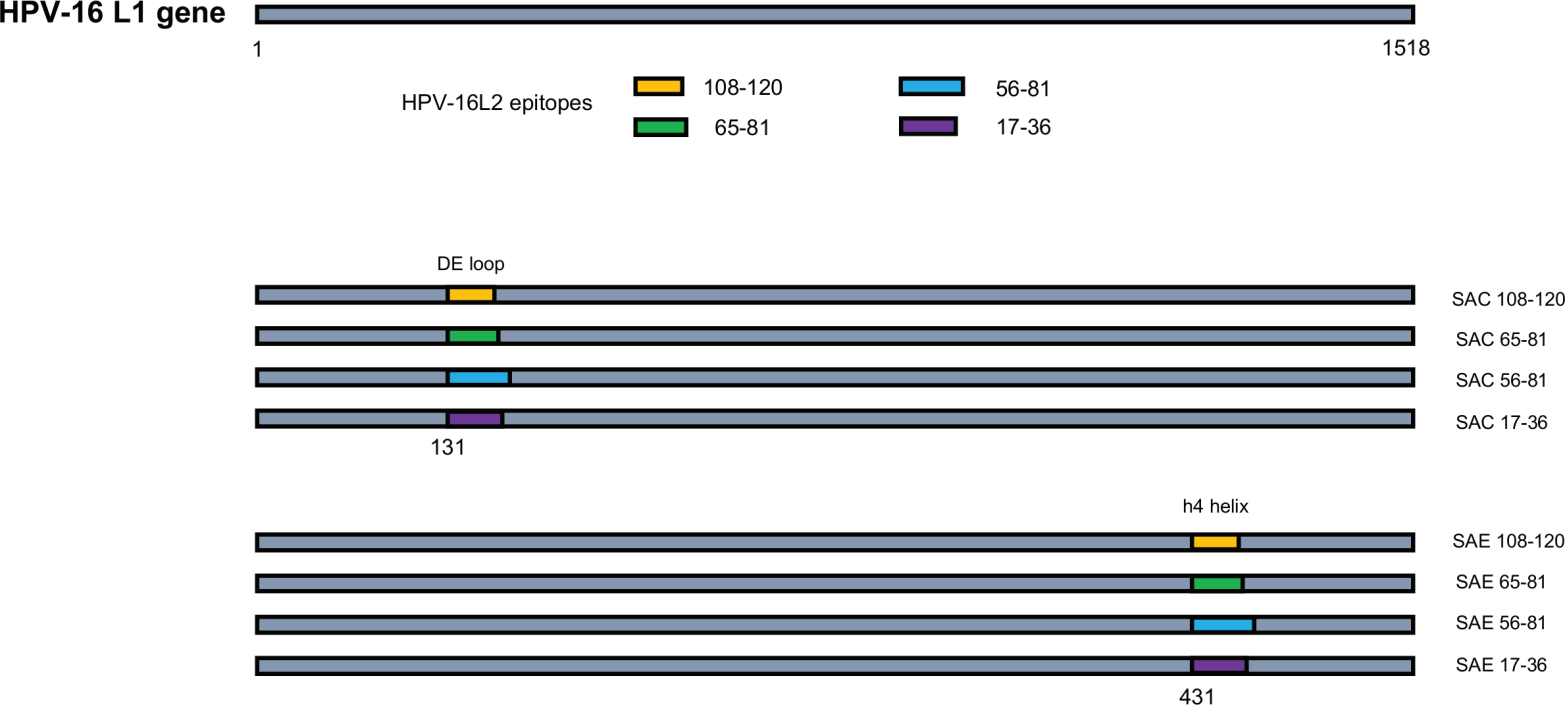
Schematic of HPV-16 L1:L2 chimera construction. HPV-16 L2 aa regions 108-120, 65-81, 56-81, and 17-36 were substituted into HPV-16 L1 at aa positions 131 in the DE loop (SAC) or 431 between the h4 and β-J structural region (SAE), to generate L1:L2 chimeras. Not drawn to scale.

The binary *Agrobacterium* vector pTRAkc-rbsc1-cTP was used to clone eight L1:L2 chimeric genes (Figure 1). Recombinant clones were transformed into *Agrobacterium tumefaciens* as described by Maclean et al. (2007). Successful transformation was confirmed by colony PCR, restriction enzyme digestion and sequencing. Starter cultures of recombinant *A. tumefaciens* pTRAkc-rbsc1-cTP SAC 108-120, SAC 65-81, SAC 56-81, SAC 17-36, SAE 65-81, hL1 (HPV-16 L1), and an empty vector (negative control) were grown at 28°C overnight in enriched Luria-Bertani broth (LBB) supplemented with 50 mg/ml carbenicillin, 30 mg/ml kanamycin, 50 mg/ml rifampicin, and 20 μM acetosyringone. The starter cultures were transferred to a bigger flask (without rifampicin) and incubated overnight. The cultures were prepared for infiltration by dilution to OD_600_ 0.5 in infiltration medium (10 mM MES, pH 5.6, 10 mM MgCl_2_, 100 μM acetosyringone). *N. benthamiana* plants (5–6 weeks old) were infiltrated with recombinant *Agrobacterium* suspensions by applying a vacuum (100 kPa) and grown for 5 days at 22°C under 16 h/8 h light/dark cycle.

### Purification of Vaccine Antigens

Whole leaves were harvested and thoroughly homogenized with a Waring-type blender in cold high-salt, low-pH extraction buffer at a w/v ratio of 1:1, supplemented with 1x Complete Mini EDTA-free protease inhibitor cocktail (Roche, Basel, Switzerland). Homogenates were incubated at 4°C with shaking for 1.5 h, filtered through four layers of Miracloth™ (Merck, Darmstadt, Germany), and clarified 2x at 10000 x *g* for 10 min at 4°C. The clarified extract was loaded onto discontinuous Optiprep^™^ (Sigma Aldrich, St Louis, MO) gradients (27, 33, 39 and 46%) and centrifuged for 3.5 h at 174500 x *g*, 15°C, in a SW 32 Ti rotor (Beckman, Brea, CA), after which 1-ml fractions were collected from the bottom of the tubes. Fractions 1–4 were pooled, added to a 5-ml ultracentrifuge tube (Ultra-Clear Thinwall TUBE, Beckman, Brea, CA) and centrifuged for 1 h at 183548 x *g* at 15°C, in a SW 55 Ti rotor (Beckman, Brea, CA). The opaque band visible after centrifugation was collected using a needle and syringe and quantified by indirect enzyme-linked immunosorbent assay (ELISA). Total L1 protein yields of the vaccine antigens was detected using Camvir-1 mAb (McLean et al., 1990): SAC 108-120 (145 mg/kg), SAC 65-81 (7.8 mg/kg), SAC 56-81 (43 mg/kg), SAC 17-36 (29 mg/kg), SAE 65-81 (1.2 mg/kg), and hL1 (142 mg/kg).

### Transmission Electron Microscopy of Purified Chimeric Virus-Like Particles

Carbon-coated copper grids (mesh size 200) were placed on a 20-μl drop of sample for 3 min and washed 5x in double distilled water. The samples were negatively stained for 1 min with 2% w/v uranyl acetate and viewed using a FEI Tecnai 20 equipped with a LaB6 emitter.

### Quantitation of Purified Chimeric Virus-Like Particles by Indirect Enzyme-Linked Immunosorbent Assay

The five L1:L2 chimeras and hL1 positive control were quantified by indirect ELISA. Ninety-six well plates (Nunc Maxisorp, ThermoFisher Scientific, Waltham, MA) were coated with: (a) 80 ng of purified HPV-16 L1 VLPs (100 μl/well) serially diluted 2-fold in coating buffer (10 mM Tris, pH 8.5) to generate a standard curve or (b) 100 μl of vaccine antigen serially diluted 2-fold from 1:50 to 1:400 in coating buffer, and incubated overnight at 4°C with gentle shaking. Plates were blocked with 300 μl of blocking buffer (1x Tris-Cl (TBS), pH 7.5, 5% non-fat dried milk) for 2 h at room temperature after which they were washed 4x with 1x TST (1x TBS, 0.05% Tween 20) wash buffer. A volume of 100 μl of Camvir-1 (1:15000) primary antibody was added to each well and the plates incubated at 37°C for 1 h. The plates were washed 4x with 1x TST, followed by the addition of 100 μl of alkaline phosphatase-conjugated anti-mouse IgG secondary antibody (1:10000) (Sigma Aldrich, St Louis, MO) to each well and incubated at 37°C for 1 h. For the final washes, plates were washed with 1x TBS (pH 9) after which 200 μl of SIGMAFAST^™^ p-nitrophenyl phosphate (Sigma Aldrich, St Louis, MO) substrate was added to each well and incubated in the dark for 30 min. The absorbance was detected at 405 nm using a Bio-Tek Powerwave XS spectrophotometer. Total L1 yield of each vaccine antigen was calculated using the average absorbance values obtained and the equation of the chart generated from the standard curve. The negative control was quantified by total soluble protein (TSP) using the Bio-Rad DC Protein Assay (Bio-Rad, Irvine, CA).

### Characterization of Chimeric Virus-Like Particle Epitope Display by Indirect Enzyme-Linked Immunosorbent Assay

One hundred nanograms (SAC 108-120, SAC 65-81, SAC 17-36 and hL1) or 50 ng (SAC and SAE 65-81) of native cVLPs or hL1 VLPs prepared in 100 μl of coating buffer were coated onto 96-well plates (Nunc Maxisorp, ThermoFisher Scientific, Waltham, MA) and incubated overnight at 4°C with gentle shaking. For denaturing conditions, cVLPs or hL1 VLPs were dried onto the 96-well plates without a lid in 0.2 M NaHCO_3_ (pH 10.6) + 0.01 M freshly added dithiothreitol (DTT) buffer overnight at 37°C. The next day, plates were blocked with 300 μl blocking buffer for 2 h at room temperature, followed by 4x washes with 1x TST wash buffer. Five-fold serial dilutions of antibodies (1:200–1:125000) in blocking buffer were added to the wells in triplicate (100 μl/well) and incubated at 37°C for 1 h. Antibodies used were neutralizing monoclonal antibodies (mAbs) H16:V5, H16.E70, H16.U4, H16.9A, H16.J4 (Christensen et al., 1996), and L2 4B4 [gift from Dr. Neil Christensen (Dept Pathology, Penn State, PA)], linear non-neutralizing commercial mAb Camvir-1, or rabbit serum raised against HPV-16 L2. Plates were then washed again and 100 μl of alkaline phosphatase-conjugated goat anti-mouse (1:10000) or goat anti-rabbit (1:5000) secondary antibody added, and incubated at 37°C for 1 h. Final washes and detection were performed as described above.

### Immunization of Mice

Animal use and care was approved by the Faculty of Health Sciences Animal Ethics Committee, University of Cape Town (FHS AEC ref.: 014/024). Forty female Balb/c mice (five mice per group) were immunized subcutaneously by injection in the left or right flank with the five plant-derived candidate cVLP vaccines: SAC 108-120, 65-81, 56-81, 17-36, and SAE 65-81, hL1 (positive HPV-16 L1 VLP control), and two negative controls (plant extract from *A. tumefaciens*-infected plants containing empty vector and PBS) (Table 1). Pre-bleed (PB) sera were collected 3 days prior to vaccination on Day 0. Mice were immunized on Day 0 and boosted with the same doses on Day 14 and Day 28, and a test bleed collected on Day 42 to ascertain if an additional boost was required. An additional boost was administered on Day 45 and final bleed (FB) sera were collected by cardiac puncture on Day 59.

**Table 1:** Vaccine group information for immunization study.

Vaccine group	Vaccine construct	Protein content	Mice per group	Antigen dose (μg)
G1	SAC 108-120	HPV-16 L1:L2 SAC 108-120	5	5
G2	SAC 65-81	HPV-16 L1:L2 SAC 65-81	5	0.8
G3	SAC 56-81	HPV-16 L1:L2 SAC 56-81	5	4.5
G4	SAC 17-36	HPV-16 L1:L2 SAC 17-36	5	2.85
G5	SAE 65-81	HPV-16 L1:L2 SAE 65-81	5	0.26
G6	hL1	HPV-16 L1	5	5
G7	Empty vector	—	5	n/a
G8	PBS	—	5	n/a

### Indirect Enzyme-Linked Immunosorbent Assay Detection of Anti-L1 Antibodies in Mouse Sera

Ninety-six-well Maxisorp plates were coated with 100 ng of purified HPV-16 L1 protein per well and incubated overnight at 4°C. Indirect ELISAs were performed as described above. FB sera were serially diluted 3-fold from 1:50 to 1:1350. All anti-L1 titers are stated as the reciprocal of the maximum dilution with higher absorbance readings than the corresponding PB serum at 1:50.

### Western Blot Detection of Anti-L2 Antibodies in Mouse Sera

HPV-16 L2 was expressed in *Escherichia coli* DH5-α using pProEx^™^-HTb (ThermoFisher Scientific, Waltham, MA). The recombinant *E. coli* culture was inoculated in 10 ml of LB, supplemented with 100 μg/ml ampicillin and incubated for 16 h at 37°C with agitation, after which it was used to inoculate 500 ml of LB medium. The culture was incubated with agitation at 37°C until it reached an A_590_ of 0.5–1.0 and induced by the addition of 0.6 mM isopropylthio-β-D-galactoside (IPTG) and the culture incubated at 37°C for 2 h. The cells were harvested by centrifugation at 10000 x *g* for 10 min. Inclusion bodies were purified from the *E. coli* cell pellet using Bugbuster® (Novagen, USA) according to the manufacturer’s instructions. Ten microliters were loaded into the wells of 10% SDS-PAGE gels, transferred to nitrocellulose membrane by semi-dry electroblotting, and strips probed with pooled sera from each vaccine group at 1:2000. Mouse anti-His mAb (Bio-Rad, Irvine, CA) was used as positive control antibody at 1:2000. Strips were then probed with alkaline phosphatase-conjugated anti-mouse IgG secondary antibody (1:10000).

### Standard L1 Pseudovirion-Based Neutralization Assay

HPV PsV production, purification, and neutralization were performed as described by Buck et al. (2005) and with a few changes as described by Pineo et al. (2013). PsVs of eight different HPV types: HPV-6, 11, 16, 18, 31, 45, 52, and 58, were produced. Sera that neutralized PsVs by at least 50% were then further titrated to determine end-point titers. Neutralization titers are stated as the reciprocal of the maximum serum dilution which reduced secreted alkaline phosphatase (SEAP) activity by >50% in comparison to the PsV only control sample, which was not treated with serum/mAb.

## Results

### Chimeric Virus-Like Particle Purification by Isopycnic Centrifugation

**Figure 2:**
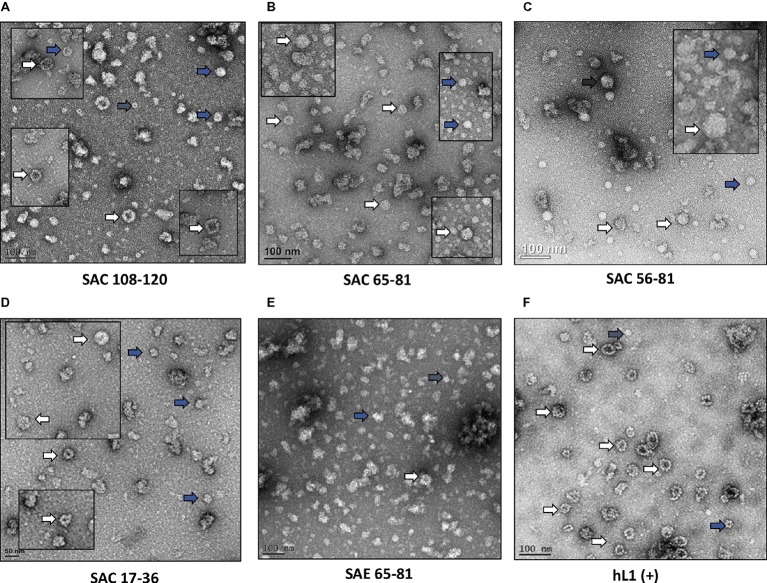
Transmission electron micrographs of purified cVLPs. Purification of cVLPs in a high-salt, low-pH buffer, followed by ultracentrifugation on a discontinuous Optiprep™ gradient. **(A)** SAC 108-120, **(B)** SAC 65-81, **(C)** SAC 56-81, **(D)** SAC 17-36, **(E)** SAE 65-81, and **(F)** HPV-16 hL1. Labels: white arrows, cVLPs 50–60 nm; blue arrows, small cVLPs 25–40 nm; grey arrows, capsomeres ~10 nm. Scale bar indicated at the bottom left of each image.

HPV-16 L2 peptides, 108-120, 65-81, 56-81, and 17-36 were substituted into HPV-16 L1 DE loop from position 131 to generate four SAC chimeras or into the C-terminal between the h4 and β-J structural region from position 431 to generate four SAE chimeras (Figure 1). Only chimeras that were shown to form higher order structures were selected for further study, namely: SAC 108-120, SAC 65-81, SAC 56-81, SAC 17-36, and SAE 65-81. HPV-16 L1:L2 cVLPs, HPV-16 hL1 VLPs, and an empty vector control were extracted and purified in a high-salt, low-pH buffer on discontinuous Optiprep™ density gradients. Purified cVLPs were visualized by TEM to determine their structural integrity prior to vaccination (Figure 2). Chimeras of SAC 108-120, SAC 65-81, SAC 56-81, and SAC 17-36 (Figures 2A–D, respectively) showed cVLPs that ranged in size from 50 to 60 nm (white arrows), small cVLPs (25–40 nm, blue arrows), and capsomeres (10 nm, grey arrows), with SAC 108-120 cVLPs (Figure 2A) being the most similar to purified HPV16-hL1 VLPs (Figure 2F). SAE 65-81 (Figure 2E) showed few cVLPs with mostly aggregates present. HPV-16 hL1 (Figure 2F) assembled into particles measuring 50–60 nm in size, with a few small VLPs present. cVLPs were comparable to other chimeras and VLPs purified previously in our group by density centrifugation (Varsani et al., 2003; Maclean et al., 2007), heparin chromatography (Pineo et al., 2013), and cation exchange chromatography (McGrath et al., 2013).

### L1 and L2 Epitope Display on the Chimeric Virus-Like Particle Scaffold

**Figure 3:**
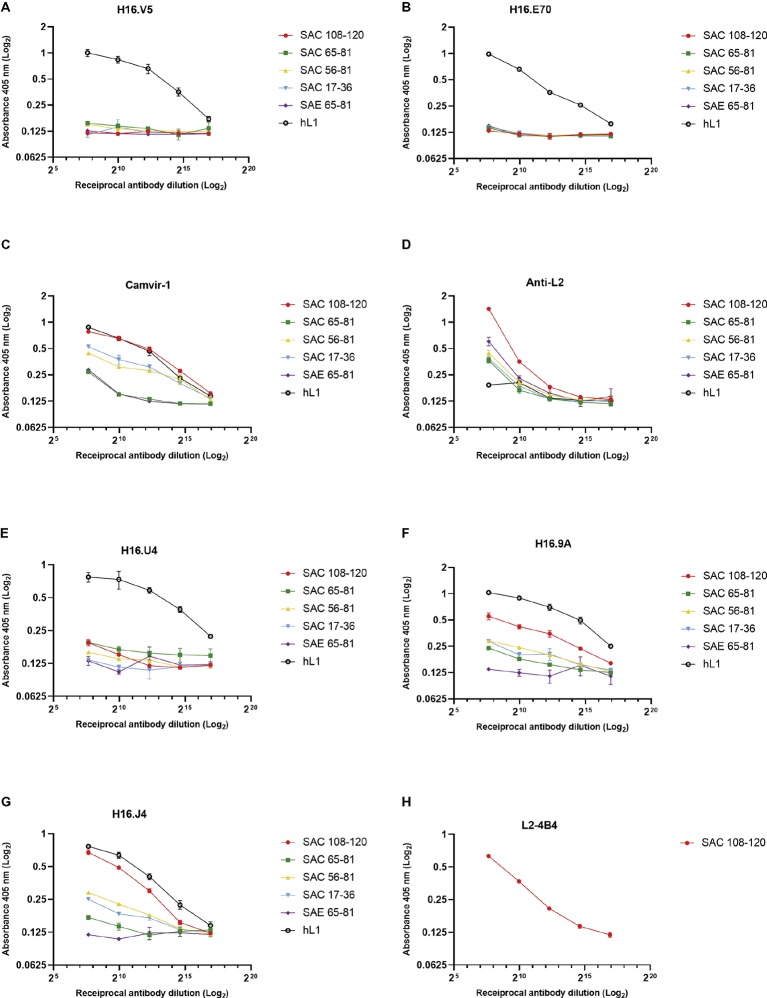
Characterization of cVLP epitope display by indirect ELISA. Binding of monoclonal and polyclonal antibodies to HPV-16 L1:L2 cVLPs and HPV-16 L1 VLPs under native conditions were analyzed in triplicate using conformational neutralizing mAbs H16.V5 **(A)**, H16.E70 **(B)**, H16.U4 **(E)**, H16.9A **(F)**, linear neutralizing mAb H16.J4 **(G)**, non-neutralizing mAb Camvir-1 **(C)**, mAb to L2 peptide 108-120 (L2-4B4) **(H)**, and polyclonal anti-L2 serum **(D)**. Error bars indicate standard deviation.

The antigenicity of cVLPs and their ability to present the substituted L2 peptides were analyzed by indirect ELISA using a panel of mAbs and anti-L2 polyclonal serum. Under native conditions (Figure 3), conformationally dependent neutralizing mAbs H16.V5 and H16.E70 did not bind the L1:L2 cVLPs (Figures 3A,B), indicating the disruption or steric hindrance of the V5 and E70 neutralizing epitopes by substitution of the L2 peptides. The anti-L2 polyclonal serum reacted with all cVLPs in native form, indicating the L2 peptides were displayed on the virion surface, with strongest binding observed for SAC 108-120 (Figure 3D). Additionally, binding of the mAb L2-4B4, which recognizes the L2 peptide 108-120 (Figure 3H), showed strong binding to SAC 108-120 cVLPs. Under denaturing conditions, binding by anti-L2 polyclonal sera and L2-4B4 mAb was slightly diminished (Supplementary Figures S1D,H). Binding was also seen for the non-neutralizing mAb Camvir-1, that recognizes a linear epitope L1 aa 204-210 (Figure 3C). As expected, native hL1 VLPs were bound by H16.V5 and H16.E70 (Figures 3A,B), but binding was diminished under denaturing conditions (Supplementary Figures S1A,B).

To further characterize if other L1 neutralizing epitopes were displayed on the cVLPs, an additional panel of mAbs were tested. Neutralizing mAbs H16.9A (conformation specific) and H16.J4 (binds linear epitope between aa 261 and 280) bound to all native SAC cVLPs (Figures 3F,G), with the highest affinity for SAC 108-120. H16.U4 however showed decreased binding for SAC 108-120, SAC 56-81, and SAC 17-36 cVLPs (Figure 3E). No binding of these mAbs to native SAE 65-81 cVLPs (Figures 3E–G) was observed, and this may be due to the poor assembly of cVLPs as observed by TEM (Figure 2E) and/or disruption or steric hindrance as mentioned above. Under denaturing conditions, mAb H16.U4 and H16.9A showed no binding to all cVLPs or hL1 VLPs (Supplementary Figures 1E,F, respectively). Only Camvir-1 and H16.J4 mAbs showed binding affinity for denatured cVLPs (Supplementary Figures S1C,G, respectively).

### Determination of Anti-L1 Titers

**Figure 4:**
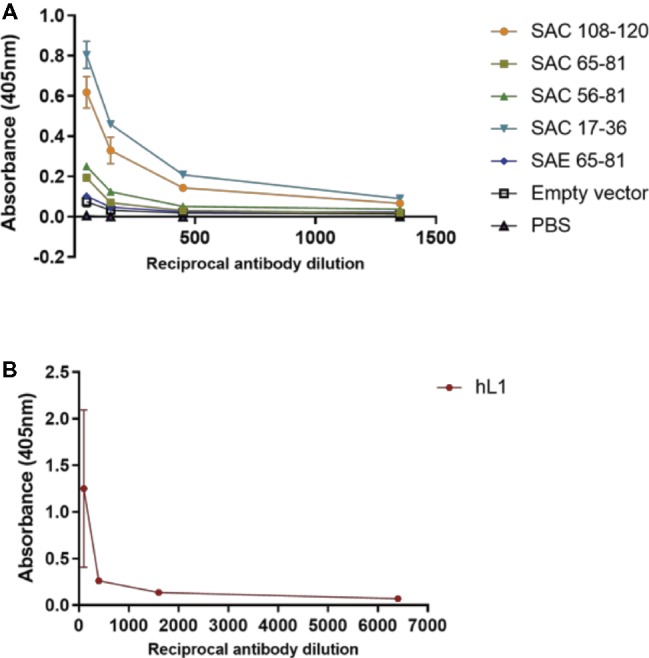
Anti-L1 titers of cVLP **(A)** and hL1 **(B)** antisera. Plates were coated with purified L1 antigen and a titration of final bleed antisera performed to determine end-point titers. Serum titers are reported for OD values greater than the mean OD of pre-bleed sera. Error bars indicate standard deviation.

Mice were subcutaneously injected with plant-purified antigens and boosted three times at 2-week intervals. Due to the yields obtained from cVLP purification, it was not possible to vaccinate mice with the desired dose of 5 μg; therefore, with the exception of SAC 108-120 and hL1 VLPs, all other chimeras were used at the maximum dose possible (Table 1). It has previously been shown that vaccine doses of 8 ng and 1 μg elicited high anti-HPV-16 L1 IgG titers and nAbs (Kim et al., 2012). Sera from individual mice were pooled and anti-L1 antibody titers determined by indirect ELISAs (Figure 4A). No anti-L1 response was detected for PB sera of all groups or for the FB of PBS negative control sera. A titer of 50 was observed for empty vector FB serum, which may be due to co-purification of plant proteins. SAC 108-120 and SAC 17-36 had anti-L1 titers of 1,350, with sera of SAC 65-81 and SAC 56-81 with titers of 150. The lowest titer of 50 was observed for SAE 65-81, similar to the titer of the empty vector. The highest anti-L1 titers of 6,400 were obtained for positive control hL1 sera (Figure 4B).

### Anti-L2 Humoral Responses

PB and FB sera for all individual mice in the different vaccine groups were pooled and analyzed for the presence of anti-L2 antibodies by western blot using *E. coli-*purified L2 as antigen (Supplementary Figure S2). Of the chimera vaccine groups, an expected band of ~80 kDa (black arrow) was detected only for SAC 108-120 FB serum and a very faint band for SAC 17-36 serum. These bands were similar in size to that of the L2 positive control. No bands were observed for the negative controls (empty vector, PBS and hL1) (Supplementary Figure S2).

### L1 Pseudovirion-Based Neutralization Assay

Purified PsVs of HPV types 6/11/16/18/31/45/52 and 58 were used in L1-PBNAs to test the ability of sera obtained from vaccinated mice to neutralize PsVs. These HPV types were chosen based on the HPVs the L2 epitopes are known to cross-neutralize. Pooled mouse sera were initially tested for neutralization at dilutions of 1:50 and 1:200 prior to titration (data not shown), and only sera that showed at least 50% neutralization of PsVs were titrated further. PB sera were only tested at a 1:50 dilution due to limited blood volume, and no neutralization was observed with PB sera (Table 2). The neutralization titers for all sera tested (Table 2) were low, except for hL1 neutralization of HPV-16 PsVs, with a titer ≥6,400. This was expected as no structural modifications were made to L1 VLPs in comparison to the chimeras tested. SAE 65-81 serum neutralized HPV-11 PsVs at a titer of 50. HPV-18 PsVs were neutralized with sera from SAC 108-120, SAC 65-81, and SAC 56-81 at titers of 200, and with SAE 65-81 serum at a titer of 50. HPV-58 PsVs had a neutralization titer of 50 for SAC 65-81 sera.

**Table 2:** Summary of *in vitro* PsV neutralization titers in L1 PBNA.

Neutralization titers								
	HPV-11		HPV-16		HPV-18		HPV-58	
	PB	FB	PB	FB	PB	FB	PB	FB
SAC 108-120	NN	NN	NN	NN	NN	200	NN	NN
SAC 65-81	NN	NN	NN	NN	NN	200	NN	50
SAC 56-81	NN	NN	NN	NN	NN	200	NN	NN
SAC 17-36	NN	NN	NN	NN	NN	NN	NN	NN
SAE 65-81	NN	100	NN	50	NN	50	NN	NN
hL1	NN	NN	NN	≥6,400	NN	NN	NN	NN
Empty vector	NN	NN	NN	NN	NN	NN	NN	NN
PBS	NN	NN	NN	NN	NN	NN	NN	NN

*Assays were performed in duplicate due to limited blood volume*.

*NN – No neutralization*.

## Discussion

L1 VLPs have successfully been expressed and purified from plants (Biemelt et al., 2003; Maclean et al., 2007; Fernandez-San et al., 2008). L1:L2 chimeras ranging in size from 50 to 60 nm have successfully been produced in insect cells and purified by ultracentrifugation on sucrose-PBS and CsCl-PBS density gradients (Varsani et al., 2003; Schellenbacher et al., 2009, 2013; Huber et al., 2015, 2017). In this study of plant-produced cVLPs, differences in cVLP assembly were observed (Figure 2). These differences may be attributed to the length and amino acid sequence of the L2 peptide used. Cys residues 175 and 428 have been shown to be critical for the formation of disulfide bonds between capsomeres for the formation of VLPs and mutations of these residues prevents the formation of VLPs (Li et al., 1998; McCarthy et al., 1998; Sapp et al., 1998; Fligge et al., 2001; Varsani et al., 2006a). Although Cys^175^ and Cys^428^ are not lost due to L2 peptide substitution, the rate of formation of disulfide bonds between neighboring L1 capsomeres may have been decreased due to a slow kinetic thio-disulfide interchange rate (Nagy, 2013). Of the four L2 peptide substitutions made, L2 108-120 is the shortest epitope, suggesting that longer epitopes may be detrimental to complete particle assembly and thus epitope display, no matter where they are substituted. This was also observed by Pineo et al. (2013) and McGrath et al. (2013) who investigated the production of cVLPs in plant and insect cell systems, respectively. In these studies, L2 peptides were substituted in the h4 helix from position 414 in the C-terminal region and although L2 108-120 substitution did not eliminate the Cys^428^ residue involved in disulfide cross-linking with Cys^175^, substitution of L2 56-81 and 17-36 did, resulting in the formation of capsomeres and aggregates. In contrast, Matic et al. (2011) produced plant-made HPV-16 L1 chimeras containing influenza virus type A M2e epitopes in the h4 helix and between the h4 and β-J region, and found that a longer epitope (23 residues) was better than a shorter epitope (eight residues) in the display of the M2e epitope. Additionally, the amino acid sequence composition can affect interaction with other residues (due to charge and size) and therefore folding. Specifically, the addition of two Cys residues in the L2 epitope 17-36 may form disulfide bonds with Cys^175^ or Cys^428^ in the L1 backbone, accounting for the particles observed in Figure 2. These data suggest that protein modeling of the interactions of substituted epitopes of varying length and sequence with L1 residues requires further investigation.

VLPs have been shown to be strongly immunogenic due to the repetitive display of epitopes on their surfaces, their interaction with antigen-presenting cells (APCs), and their activation of B cells (Chackerian et al., 2008; Schiller and Lowy, 2018). Several L2 aa epitopes have been inserted by others into L1 loops for the generation of cVLPs. Insertion of L2 aa 17-36, 28-31, 35-75, 69-81, 108-120, and 115-15 into BPV-1 L1 DE loop at position 133/134, elicited anti-L1 and anti-L2 responses in mice (Slupetzky et al., 2007; Schellenbacher et al., 2009). Additionally, substitution (from position 131) of L2 aa 108-120 into the HPV-16 DE loop of insect-cell produced chimeras (Varsani et al., 2003), or L2 aa 108-120, 56-81, and 17-36 in the h4 helix (from position 414) of plant-produced (Pineo et al., 2013) or insect cell-produced (McGrath et al., 2013) chimeras, also elicited anti-L1 and anti-L2 responses in mice. The ability of antibodies obtained from sera to neutralize PsVs was investigated in PBNAs. Neutralization of homologous HPV-16 PsVs was only observed with antisera of SAE 65-81 at a titer of 50 and hL1 VLPs at titer ≥6,400 (Table 2). The titer observed for hL1 was similar to nAb titers obtained in other studies testing plant-produced chimeras or VLPs: 500–5,000 (Pineo et al., 2013); 6,400 (Maclean et al., 2007); 400 (Paz De la et al., 2009). The low titer obtained by SAE 65–81, in addition to the low vaccination dose, may be due to partially formed cVLPs (Figure 2E) and the presentation of L2 on the capsid. Position 431 is located in the C-terminal arm of L1 and is not directly involved in the correct folding of VLPs, but it is close to the h4 helix region where residues 414–426 play a role in VLP assembly (Varsani et al., 2003; Bishop et al., 2007). Steric hindrance due to the substitution of residues with different charges may therefore affect correct folding. No detectable nAbs to HPV-16 were elicited by SAC 108-120, SAC 65-81, SAC 56-81, and SAC 17-36 despite forming cVLPs, suggesting that antigen bound by sera in indirect ELISA and L2 western blots (Figure 4, Supplementary Figure S2) were detected by non-neutralizing antibodies. Cross-neutralization of heterologous HPV PsV types was observed with neutralization of HPV-18 by SAC 108-120, SAC 65-81, SAC 56-81, and SAE 65-81 antisera. HPV-58 was cross-neutralized by SAC 108-120, and HPV-11 by SAE 65-81 antisera. All neutralizing titers observed were between 50 and 200 (Table 2). Unexpectedly, SAC 17-36 did not show any cross-neutralization despite this epitope being shown by Schellenbacher et al. (2013) to elicit robust anti-L2 antibodies and cross-neutralize up to 16 high-risk HPVs. Moreover, in L2-specific PBNAs (performed as described by Day et al. (2012)), no nAb titers were observed for all antisera tested. L2 PBNAs previously performed by our group with sera from plant-produced HPV-16 L1:L2 chimeras (L2 substituted in the h4 helix (Pineo et al., 2013)) showed low cross-neutralization, with nAb titers of 50 for HPV-11 (L1:L2 56-81) and − 18 (L1:L2 17-36), but no neutralization to homologous HPV-16 PsVs (unpublished data). Surprisingly, no L2 nAb titers were detected for L1:L2 108-120, even though it was found to be the best candidate vaccine as it elicited nAbs to HPV-16 and HPV-52 in L1 PBNAs (Pineo et al., 2013). These same chimeras produced in insect cells (McGrath et al., 2013) elicited sera that showed neutralization of HPV-16/18/31/52 in L2 PBNAs (unpublished data), suggesting that plant-produced chimeras may not assemble as efficiently and thus not display L2 epitopes as well. It is possible this may be the case as it has been suggested that VLP assembly is sensitive to cell type (Li et al., 1997). Overall, these data show that presentation of L2 epitopes on the plant-made L1 chimera surface was not sufficient to produce potent anti-L2 nAbs that are protective against multiple oncogenic HPV types in neutralization assays.

There are several possible explanations for why the anti-L1 responses and L1 and L2 nAb titers were lower or not observed than has previously been reported. The display of L2 epitopes in L1 loops should preserve the L1 epitopes critical for binding by mAbs. The mAb H16.V5 binding site is a major immunodominant epitope used for the assessment of integrity and antigenicity of VLPs. It has been shown to block the binding of >70% human sera (Roden et al., 1997; Wang et al., 2003) and recognizes aa 266-297 in the FG loop and aa 339-365 in the HI loop (Christensen et al., 2001). mAbs H16:V5 and H16:E70 have been extensively mapped and residues Phe^50^, Ala^266^, and Ser^282^ of L1 are vital for binding and the generation of potent nAbs (White et al., 1999). The residues of the DE loop (aa 110-149) are not predicted to have any impact on Phe^50^, Ala^266^, and Ser^282^ residues suggesting that substitution of L2 epitopes in this region should not affect H16:V5 epitope display. However, Lee et al. (2015) have recently shown that the BC (aa 181 and 184) and DE (aa 138-141) loops contribute to binding by H16.V5, with a few contact residues in the EF and HI loops. Furthermore, Bissett et al. (2016) showed that L1 epitopes necessary for the generation of cross-neutralizing antibodies are present in the DE and FG loops. The type-specific nature of L1 nAbs is due the variation found within the L1 surface loops of different HPV genotypes (Chen et al., 2000a; Carter et al., 2003). The exposed surface loops, e.g., BC and EF, show more sequence heterogeneity than the core loops, e.g., DE, seen from analysis of intra- and inter-genotype amino acid variability (Bissett et al., 2014). This variability is thought to be a mechanism in which the virus can avoid nAbs. Tyr^135^ and Val^141^ have also been shown to be critical for binding by mAb 26D1 (Xia et al., 2016), further supporting the importance of the DE loop as a cross-neutralizing epitope. Substitution at position 131 thereby replaced regions of L1 in the DE loop that have been shown to be critical epitopes for binding by mAbs and cross-neutralizing antibodies. Huber et al. (2017) have recently shown that sequence replacement of HPV-5 L1 (aa 132-145) with HPV-17 RG1 (L2 aa 14-33) resulted in low type-specific neutralizing titers to HPV-5 and antiserum was not protective against PsV challenge *in vivo*. The authors postulated that replacement of the DE loop resulted in steric hindrance of the major HPV-5 L1 neutralization epitope(s). The four SAC chimeras (108-120, 65-81, 56-81, and 17-36) assembled into cVLPs, but showed low anti-L1 titers and low nAb titers in PBNAs, potentially as a result of the disruption of the abovementioned residues in the DE loop. Additionally, although SAE 65-81 was the only candidate vaccine that neutralized homologous HPV-16, due to the disulfide bonds between Cys^175^ and Cys^428^, residues 433-443 are less accessible (Varsani et al., 2003) and therefore presentation of the L2 peptides may not have been efficient to elicit anti-L2 antibodies.

In summary, all chimeric vaccine candidates were immunogenic. Only sera from cVLPs SAC 108-120, SAC 65-81, SAC 56-81 neutralized HPV-18 PsVs at 1:200 dilutions, with PsVs of HPV-11, HPV-16, and HPV-58 neutralized at very low dilutions in L1 PBNAs. Unexpectedly, antisera did not neutralize PsVs in L2 PBNAs, despite L2 being displayed on the L1 capsid. It is important to consider L1 neutralizing epitopes when determining the display position of L2 peptides. Although L2 substitutions did not seem to drastically affect the assembly of cVLPs, misassembled or disrupted VLPs expose epitopes with limited HPV type specificity (Christensen et al., 1994, 1996). This study highlights the importance of particle assembly, peptide presentation, and yield – factors that need to be further investigated to achieve success with next-generation HPV vaccines that elicit potent anti-L1 and anti-L2 nAbs against oncogenic HPV types.

## Ethics Statement

This study was carried out in accordance with the recommendation of the Faculty of Health Sciences Animal Ethics Committee of the University of Cape Town. The protocol was approved by the Faculty of Health Sciences Animal Ethics Committee, University of Cape Town (FHS AEC ref.: 014/024).

## Author Contributions

AC performed all experiments and wrote the manuscript. AZ supervised the project and assisted in purification experiments. ER and IH designed and conceptualized the study.

### Conflict of Interest Statement

The authors declare that the research was conducted in the absence of any commercial or financial relationships that could be construed as a potential conflict of interest.

## Supplementary Material

Supplementary Figure 1 Characterization of cVLP epitope display by indirect ELISA. Binding of monoclonal and polyclonal antibodies to HPV-16 L1:L2 cVLPs and HPV-16 L1 VLPs under denaturing conditions were analyzed in triplicate using conformational neutralizing mAbs H16.V5 **(A)**, H16.E70 **(B)**, H16.U4 **(E)**, H16.9A **(F)**, linear neutralizing mAb H16.J4 **(G)**, non-neutralizing mAb Camvir-1 **(C)**, mAb to L2 peptide 108-120 (L2-4B4) **(H)**, and polyclonal anti-L2 serum **(D)**. Error bars indicate standard deviation.

Supplementary Figure 2 Anti-L2 western blots using pooled mouse sera. HPV-16 L2 protein was probed with pre-bleed (PB) or final bleed (FB) sera at 1:2000 from the 8 vaccine groups. A band at 80 kDa is expected for L2 protein. Labels: M, molecular weight marker (kDa); +, L2 positive control detected with anti-His mAb (1:2000).

## References

[ref1] Abubakar I. Tillmann T. Banerjee A. (2015). Global, regional, and national age-sex specific all-cause and cause-specific mortality for 240 causes of death, 1990-2013: a systematic analysis for the global burden of disease study 2013. Lancet 385, 117–171. 10.1016/S0140-6736(14)61682-2 25530442 PMC4340604

[ref2] Alphs H. H. Gambhira R. Karanam B. Roberts J. N. Jagu S. Schiller J. T. (2008). Protection against heterologous human papillomavirus challenge by a synthetic lipopeptide vaccine containing a broadly cross-neutralizing epitope of L2. Proc. Natl. Acad. Sci. USA 105, 5850–5855. 10.1073/pnas.0800868105 18413606 PMC2299222

[ref3] Biemelt S. Sonnewald U. Galmbacher P. Willmitzer L. Muller M. (2003). Production of human papillomavirus type 16 virus-like particles in transgenic plants. J. Virol. 77, 9211–9220. 10.1128/JVI.77.17.9211-9220.2003, PMID: 12915537 PMC187377

[ref4] Bishop B. Dasgupta J. Chen X. S. (2007). Structure-based engineering of papillomavirus major capsid L1: controlling particle assembly. Virol. J. 4, 3–8. 10.1186/1743-422X-4-3, PMID: 17210082 PMC1781933

[ref5] Bissett S. L. Godi A. Beddows S. (2016). The DE and FG loops of the HPV major capsid protein contribute to the epitopes of vaccine-induced cross-neutralising antibodies. Sci. Rep. 6:39730. 10.1038/srep39730, PMID: 28004837 PMC5177933

[ref6] Bissett S. L. Mattiuzzo G. Draper E. Godi A. Wilkinson D. E. Minor P.. (2014). Pre-clinical immunogenicity of human papillomavirus alpha-7 and alpha-9 major capsid proteins. Vaccine 32, 6548–6555. 10.1016/j.vaccine.2014.07.116, PMID: 25203446 PMC4228199

[ref7] Bray F. Ferlay J. Soerjomataram I. Siegel R. L. Torre L. A. Jemal A. (2018). Global cancer statistics 2018: GLOBOCAN estimates of incidence and mortality worldwide for 36 cancers in 185 countries. CA Cancer J. Clin. 1–31. 10.3322/caac.21492 30207593

[ref8] Buchman G. W. Howard B. P. Abdallah N. Medicherla B. Fisher M. A. White J. M. (2016). Abstract 2367: cGMP production of a chimeric virus-like particle vaccine for prevention of HPV-associated cancers. Cancer Res. 76:2367. 10.1158/1538-7445.AM2016-2367

[ref9] Buck C. B. Cheng N. Thompson C. D. Lowy D. R. Steven A. C. Schiller J. T.. (2008). Arrangement of L2 within the papillomavirus capsid. J. Virol. 82, 5190–5197. 10.1128/JVI.02726-07, PMID: 18367526 PMC2395198

[ref10] Buck C. B. Pastrana D. V. Lowy D. R. Schiller J. T. (2005). Generation of HPV pseudovirions using transfection and their use in neutralization assays. Methods Mol. Med. 119, 445–462. 10.1385/1-59259-982-6:445, PMID: 16350417

[ref11] Cardona C. E. M. García-Perdomo H. A. J. R. P.d. S. P. (2018). Incidence of penile cancer worldwide: systematic review and meta-analysis. Pan Am. J. Public Health 41:e117. 10.26633/rpsp.2017.117 PMC6645409 31384255

[ref12] Carter J. J. Wipf G. C. Benki S. F. Christensen N. D. Galloway D. A. (2003). Identification of a human papillomavirus type 16-specific epitope on the C-terminal arm of the major capsid protein L1. J. Virol. 77, 11625–11632. 10.1128/JVI.77.21.11625-11632.2003, PMID: 14557648 PMC229369

[ref13] Casini G. L. Graham D. Heine D. Garcea R. L. Wu D. T. (2004). In vitro papillomavirus capsid assembly analyzed by light scattering. Virology 325, 320–327. 10.1016/j.virol.2004.04.034, PMID: 15246271

[ref14] Chackerian B. Durfee M. R. Schiller J. T. (2008). Virus-like display of a neo-self antigen reverses B cell anergy in a B cell receptor transgenic mouse model. J. Immunol. 180, 5816–5825. 10.4049/jimmunol.180.9.5816, PMID: 18424700 PMC3493123

[ref15] Chen X. S. Garcea R. L. Goldberg I. Casini G. Harrison S. C. (2000b). Structure of small virus-like particles assembled from the L1 protein of human papillomavirus 16. Mol. Cell 5, 557–567. 10.1016/S1097-2765(00)80449-9 10882140

[ref16] Chen C. H. Wang T. L. Hung C. F. Yang Y. Young R. A. Pardoll D. M. (2000a). Enhancement of DNA vaccine potency by linkage of antigen gene to an HSP70 gene. Cancer Res. 60, 1035–1042. 10706121

[ref17] Chen X. Zhang T. Liu H. Hao Y. Liao G. Xu X. (2018). Displaying 31RG-1 peptide on the surface of HPV16 L1 by use of a human papillomavirus chimeric virus-like particle induces cross-neutralizing antibody responses in mice. Hum. Vaccin. Immunother. 14, 2025–2033. 10.1080/21645515.2018.1464355, PMID: 29683766 PMC6149973

[ref18] Christensen N. D. Cladel N. M. Reed C. A. Budgeon L. R. Embers M. E. Skulsky D. M.. (2001). Hybrid papillomavirus L1 molecules assemble into virus-like particles that reconstitute conformational epitopes and induce neutralizing antibodies to distinct HPV types. Virology 291, 324–334. 10.1006/viro.2001.1220, PMID: 11878901

[ref19] Christensen N. D. Dillner J. Eklund C. Carter J. J. Wipf G. C. Reed C. A.. (1996). Surface conformational and linear epitopes on HPV-16 and HPV-18 L1 virus-like particles as defined by monoclonal antibodies. Virology 223, 174–184. 10.1006/viro.1996.0466, PMID: 8806551

[ref20] Christensen N. D. Höpfl R. DiAngelo S. L. Cladel N. M. Patrick S. D. Welsh P. A.. (1994). Assembled baculovirus-expressed human papillomavirus type 11 L1 capsid protein virus-like particles are recognized by neutralizing monoclonal antibodies and induce high titres of neutralizing antibodies. J. Gen. Virol. 75, 2271–2276. 10.1099/0022-1317-75-9-2271, PMID: 7521393

[ref21] Conway M. Meyers C. (2009). Replication and assembly of human papillomaviruses. J. Dent. Res. 88, 307–317. 10.1177/0022034509333446, PMID: 19407149 PMC3317948

[ref22] Day P. M. Pang Y.-Y. S. Kines R. C. Thompson C. D. Lowy D. R. Schiller J. T. (2012). A human papillomavirus (HPV) in vitro neutralization assay that recapitulates the in vitro process of infection provides a sensitive measure of HPV L2 infection-inhibiting antibodies. Clin. Vaccine Immunol. 19, 1075–1082. 10.1128/CVI.00139-12, PMID: 22593236 PMC3393370

[ref23] De Martel C. Ferlay J. Franceschi S. Vignat J. Bray F. Forman D.. (2012). Global burden of cancers attributable to infections in 2008: a review and synthetic analysis. Lancet Oncol. 13, 607–615. 10.1016/S1470-2045(12)70137-7, PMID: 22575588

[ref24] de Sanjose S. Quint W. G. Alemany L. Geraets D. T. Klaustermeier J. E. Lloveras B.. (2010). Human papillomavirus genotype attribution in invasive cervical cancer: a retrospective cross-sectional worldwide study. Lancet Oncol. 11, 1048–1056. 10.1016/S1470-2045(10)70230-8, PMID: 20952254

[ref25] de Villiers E. M. Fauquet C. Broker T. R. Bernard H. U. zur H. H. (2004). Classification of papillomaviruses. Virology 324, 17–27. 10.1016/j.virol.2004.03.033, PMID: 15183049

[ref26] Fernandez-San M. A. Ortigosa S. M. Hervas-Stubbs S. Corral-Martinez P. Segui-Simarro J. M. Gaetan J.. (2008). Human papillomavirus L1 protein expressed in tobacco chloroplasts self-assembles into virus-like particles that are highly immunogenic. Plant Biotechnol. J. 6, 427–441. 10.1111/j.1467-7652.2008.00338.x, PMID: 18422886

[ref27] Fischer R. Stoger E. Schillberg S. Christou P. Twyman R. M. (2004). Plant-based production of biopharmaceuticals. Curr. Opin. Plant Biol. 7, 152–158. 10.1016/j.pbi.2004.01.007, PMID: 15003215

[ref28] Fligge C. Giroglou T. Streeck R. E. Sapp M. (2001). Induction of type-specific neutralizing antibodies by capsomeres of human papillomavirus type 33. Virology 283, 353–357. 10.1006/viro.2000.0875, PMID: 11336560

[ref29] Gambhira R. Karanam B. Jagu S. Roberts J. N. Buck C. B. Bossis I.. (2007). A protective and broadly cross-neutralizing epitope of human papillomavirus L2. J. Virol. 81, 13927–13931. 10.1128/JVI.00936-07, PMID: 17928339 PMC2168823

[ref30] Goodman M. T. Shvetsov Y. B. McDuffie K. Wilkens L. R. Zhu X. Thompson P. J.. (2008). Prevalence, acquisition, and clearance of cervical human papillomavirus infection among women with normal cytology: Hawaii human papillomavirus cohort study. Cancer Res. 68, 8813–8824. 10.1158/0008-5472.CAN-08-1380, PMID: 18974124 PMC2727731

[ref31] Hagensee M. E. Yaegashi N. Galloway D. A. (1993). Self-assembly of human papillomavirus type 1 capsids by expression of the L1 protein alone or by coexpression of the L1 and L2 capsid proteins. J. Virol. 67, 315–322. PMID: 8380079 10.1128/jvi.67.1.315-322.1993 PMC237365

[ref32] Huber B. Schellenbacher C. Jindra C. Fink D. Shafti-Keramat S. Kirnbauer R. (2015). A chimeric 18L1-45RG1 virus-like particle vaccine cross-protects against oncogenic Alpha-7 human papillomavirus types. PLoS One 10:e0120152. 10.1371/journal.pone.0120152, PMID: 25790098 PMC4366228

[ref33] Huber B. Schellenbacher C. Shafti-Keramat S. Jindra C. Christensen N. Kirnbauer R. (2017). Chimeric L2-based virus-like particle (VLP) vaccines targeting cutaneous human papillomaviruses (HPV). PLoS One 12:e0169533. 10.1371/journal.pone.0169533 28056100 PMC5215943

[ref34] Huh W. K. Joura E. A. Giuliano A. R. Iversen O.-E. de Andrade R. P. Ault K. A.. (2017). Final efficacy, immunogenicity, and safety analyses of a nine-valent human papillomavirus vaccine in women aged 16–26 years: a randomised, double-blind trial. Lancet 390, 2143–2159. 10.1016/S0140-6736(17)31821-4, PMID: 28886907

[ref35] Jagu S. Kwak K. Karanam B. Huh W. K. Damotharan V. Chivukula S. V.. (2013). Optimization of multimeric human papillomavirus L2 vaccines. PLoS One 8:e55538. 10.1371/journal.pone.0055538, PMID: 23383218 PMC3561222

[ref36] Kawana K. Matsumoto K. Yoshikawa H. Taketani Y. Kawana T. Yoshiike K.. (1998). A surface immunodeterminant of human papillomavirus type 16 minor capsid protein L2. Virology 245, 353–359. 10.1006/viro.1998.9168, PMID: 9636375

[ref37] Kawana K. Yoshikawa H. Taketani Y. Yoshiike K. Kanda T. (1999). Common neutralization epitope in minor capsid protein L2 of human papillomavirus types 16 and 6. J. Virol. 73, 6188–6190. PMID: 10364381 10.1128/jvi.73.7.6188-6190.1999 PMC112690

[ref38] Kim H. J. Lim S. J. Kwag H.-L. Kim H.-J. (2012). The choice of resin-bound ligand affects the structure and immunogenicity of column-purified human papillomavirus type 16 virus-like particles. PLoS One 7:e35893. 10.1371/journal.pone.0052973, PMID: 22563414 PMC3338541

[ref39] Kines R. C. Thompson C. D. Lowy D. R. Schiller J. T. Day P. M. (2009). The initial steps leading to papillomavirus infection occur on the basement membrane prior to cell surface binding. Proc. Natl. Acad. Sci. 106, 20458–20463. 10.1073/pnas.0908502106, PMID: 19920181 PMC2787115

[ref40] Kirnbauer R. Booy F. Cheng N. Lowy D. R. Schiller J. T. (1992). Papillomavirus L1 major capsid protein self-assembles into virus-like particles that are highly immunogenic. Proc. Natl. Acad. Sci. USA 89, 12180–12184. 1334560 10.1073/pnas.89.24.12180 PMC50722

[ref41] Kohl T. Hitzeroth I. I. Stewart D. Varsani A. Govan V. A. Christensen N. D.. (2006). Plant-produced cottontail rabbit papillomavirus L1 protein protects against tumor challenge: a proof-of-concept study. Clin. Vaccine Immunol. 13, 845–853. 10.1128/CVI.00072-06, PMID: 16893983 PMC1539125

[ref42] Kondo K. Ishii Y. Ochi H. Matsumoto T. Yoshikawa H. Kanda T. (2007). Neutralization of HPV16, 18, 31, and 58 pseudovirions with antisera induced by immunizing rabbits with synthetic peptides representing segments of the HPV16 minor capsid protein L2 surface region. Virology 358, 266–272. 10.1016/j.virol.2006.08.037, PMID: 17010405

[ref43] Kondo K. Ochi H. Matsumoto T. Yoshikawa H. Kanda T. (2008). Modification of human papillomavirus-like particle vaccine by insertion of the cross-reactive L2-epitopes. J. Med. Virol. 80, 841–846. 10.1002/jmv.21124, PMID: 18360909

[ref44] Lee H. Brendle S. A. Bywaters S. M. Guan J. Ashley R. E. Yoder J. D.. (2015). A cryo-electron microscopy study identifies the complete H16. V5 epitope and reveals global conformational changes initiated by binding of the neutralizing antibody fragment. J. Virol. 89, 1428–1438. 10.1128/JVI.02898-14, PMID: 25392224 PMC4300654

[ref45] Li M. Beard P. Estes P. A. Lyon M. K. Garcea R. L. (1998). Intercapsomeric disulfide bonds in papillomavirus assembly and disassembly. J. Virol. 72, 2160–2167. PMID: 9499072 10.1128/jvi.72.3.2160-2167.1998 PMC109511

[ref46] Li M. Cripe T. P. Estes P. A. Lyon M. K. Rose R. C. Garcea R. L. (1997). Expression of the human papillomavirus type 11 L1 capsid protein in *Escherichia coli*: characterization of protein domains involved in DNA binding and capsid assembly. J. Virol. 71, 2988–2995. 9060658 10.1128/jvi.71.4.2988-2995.1997 PMC191427

[ref47] Lowe J. Panda D. Rose S. Jensen T. Hughes W. A. Tso F. Y.. (2008). Evolutionary and structural analyses of alpha-papillomavirus capsid proteins yields novel insights into L2 structure and interaction with L1. Virol. J. 5:150. 10.1186/1743-422X-5-150, PMID: 19087355 PMC2630942

[ref48] Maclean J. Koekemoer M. Olivier A. J. Stewart D. Hitzeroth I. I. Rademacher T.. (2007). Optimization of human papillomavirus type 16 (HPV-16) L1 expression in plants: comparison of the suitability of different HPV-16 L1 gene variants and different cell-compartment localization. J. Gen. Virol. 88, 1460–1469. 10.1099/vir.0.82718-0, PMID: 17412974

[ref49] Matic S. Rinaldi R. Masenga V. Noris E. (2011). Efficient production of chimeric human papillomavirus 16 L1 protein bearing the M2e influenza epitope in Nicotiana benthamiana plants. BMC Biotechnol. 11. 10.1186/1472-6750-11-106, PMID: 22085463 PMC3248878

[ref50] McCarthy M. P. White W. I. Palmer-Hill F. Koenig S. Suzich J. A. (1998). Quantitative disassembly and reassembly of human papillomavirus type 11 virus like particles in vitro. J. Virol. 72, 32–41. PMID: 9420197 10.1128/jvi.72.1.32-41.1998 PMC109346

[ref51] McGrath M. de Villiers G. K. Shephard E. Hitzeroth I. I. Rybicki E. P. (2013). Development of human papillomavirus chimaeric L1/L2 candidate vaccines. Arch. Virol. 158, 2079–2088. 10.1007/s00705-013-1713-8 23636405

[ref52] McLean C. S. Churcher M. J. Meinke J. Smith G. L. Higgins G. Stanley M.. (1990). Production and characterisation of a monoclonal antibody to human papillomavirus type 16 using recombinant vaccinia virus. J. Clin. Pathol. 43, 488–492. 10.1136/jcp.43.6.488, PMID: 2166093 PMC502503

[ref53] Modis Y. Trus B. L. Harrison S. C. (2002). Atomic model of the papillomavirus capsid. EMBO J. 21, 4754–4762. 10.1093/emboj/cdf494, PMID: 12234916 PMC126290

[ref54] Nagy P. (2013). Kinetics and mechanisms of thiol–disulfide exchange covering direct substitution and thiol oxidation-mediated pathways. Antioxid. Redox Signal. 18, 1623–1641. 10.1089/ars.2012.4973, PMID: 23075118 PMC3613173

[ref55] Naud P. S. Roteli-Martins C. M. De Carvalho N. S. Teixeira J. C. de Borba P. C. Sanchez N.. (2014). Sustained efficacy, immunogenicity, and safety of the HPV-16/18 AS04-adjuvanted vaccine: final analysis of a long-term follow-up study up to 9.4 years post-vaccination. Hum. Vaccin. Immunother. 10, 2147–2162. 10.4161/hv.29532, PMID: 25424918 PMC4896780

[ref56] Parkin D. M. Bray F. (2006). The burden of HPV-related cancers. Vaccine 24, S11–S25. 10.1016/j.vaccine.2006.05.111, PMID: 16949997

[ref57] Pastrana D. V. Gambhira R. Buck C. B. Pang Y. Y. Thompson C. D. Culp T. D.. (2005). Cross-neutralization of cutaneous and mucosal papillomavirus types with anti-sera to the amino terminus of L2. Virology 337, 365–372. 10.1016/j.virol.2005.04.011, PMID: 15885736

[ref58] Paz De la R. G. Monroy-Garcia A. Mora-Garcia M. L. Pena C. G. Hernandez-Montes J. Weiss-Steider B.. (2009). An HPV 16 L1-based chimeric human papilloma virus-like particles containing a string of epitopes produced in plants is able to elicit humoral and cytotoxic T-cell activity in mice. Virol. J. 6, 2–12. 10.1186/1743-422X-6-2, PMID: 19126233 PMC2639544

[ref59] Pineo C. B. Hitzeroth I. I. Rybicki E. P. (2013). Immunogenic assessment of plant-produced human papillomavirus type 16 L1/L2 chimaeras. Plant Biotechnol. J. 11, 964–975. 10.1111/pbi.12089, PMID: 23924054

[ref60] Regnard G. L. Halley-Stott R. P. Tanzer F. L. Hitzeroth I. I. Rybicki E. P. (2010). High level protein expression in plants through the use of a novel autonomously replicating geminivirus shuttle vector. Plant Biotechnol. J. 8, 38–46. 10.1111/j.1467-7652.2009.00462.x, PMID: 19929900

[ref61] Roden R. B. Armstrong A. Haderer P. Christensen N. D. Hubbert N. L. Lowy D. R.. (1997). Characterization of a human papillomavirus type 16 variant-dependent neutralizing epitope. J. Virol. 71, 6247–6252. PMID: 9223527 10.1128/jvi.71.8.6247-6252.1997 PMC191893

[ref62] Roden R. B. S. Stern P. L. (2018). Opportunities and challenges for human papillomavirus vaccination in cancer. Nat. Rev. Cancer 18, 240–254. 10.1038/nrc.2018.13, PMID: 29497146 PMC6454884

[ref63] Roden R. B. Yutzy W. H. Fallon R. Inglis S. Lowy D. R. Schiller J. T. (2000). Minor capsid protein of human genital papillomaviruses contains subdominant, cross-neutralizing epitopes. Virology 270, 254–257. 10.1006/viro.2000.0272, PMID: 10792983

[ref64] Rosa M. I. Fachel J. M. Rosa D. D. Medeiros L. R. Igansi C. N. Bozzetti M. C. (2008). Persistence and clearance of human papillomavirus infection: a prospective cohort study. Am. J. Obstet. Gynecol. 199, 617. e611–617. e617. 10.1097/COH.0b013e3282fbaaa7, PMID: 18799155

[ref65] Rybicki E. P. (2010). Plant-made vaccines for humans and animals. Plant Biotechnol. J. 8, 620–637. 10.1111/j.1467-7652.2010.00507.x, PMID: 20233333 PMC7167690

[ref66] Sadeyen J.-R.é. Tourne S. Shkreli M. Sizaret P.-Y. Coursaget P. (2003). Insertion of a foreign sequence on capsid surface loops of human papillomavirus type 16 virus-like particles reduces their capacity to induce neutralizing antibodies and delineates a conformational neutralizing epitope. Virology 309, 32–40. 10.1016/S0042-6822(02)00134-4, PMID: 12726724

[ref67] Sapp M. Fligge C. Petzak I. Harris J. R. Streeck R. E. (1998). Papillomavirus assembly requires trimerization of the major capsid protein by disulfides between two highly conserved cysteines. J. Virol. 72, 6186–6189. PMID: 9621087 10.1128/jvi.72.7.6186-6189.1998 PMC110432

[ref68] Schellenbacher C. Kwak K. Fink D. Shafti-Keramat S. Huber B. Jindra C.. (2013). Efficacy of RG1-VLP vaccination against infections with genital and cutaneous human papillomaviruses. J. Investig. Dermatol. 133, 2706–2713. 10.1038/jid.2013.253, PMID: 23752042 PMC3826974

[ref69] Schellenbacher C. Roden R. Kirnbauer R. (2009). Chimeric L1-L2 virus-like particles as potential broad-spectrum human papillomavirus vaccines. J. Virol. 83, 10085–10095. 10.1128/JVI.01088-09, PMID: 19640991 PMC2748020

[ref70] Schellenbacher C. Roden R. B. S. Kirnbauer R. (2017). Developments in L2-based human papillomavirus (HPV) vaccines. Virus Res. 231, 166–175. 10.1016/j.virusres.2016.11.020, PMID: 27889616 PMC5549463

[ref71] Schiller J. Lowy D. (2018). Explanations for the high potency of HPV prophylactic vaccines. Vaccine 36, 4768–4773. 10.1016/j.vaccine.2017.12.079, PMID: 29325819 PMC6035892

[ref72] Slupetzky K. Gambhira R. Culp T. D. Shafti-Keramat S. Schellenbacher C. Christensen N. D.. (2007). A papillomavirus-like particle (VLP) vaccine displaying HPV16 L2 epitopes induces cross-neutralizing antibodies to HPV11. Vaccine 25, 2001–2010. 10.1016/j.vaccine.2006.11.049, PMID: 17239496 PMC3935451

[ref73] Slupetzky K. Shafti-Keramat S. Lenz P. Brandt S. Grassauer A. Sara M. (2001). Chimeric papillomavirus-like particles expressing a foreign epitope on capsid surface loops. J. Gen. Virol. 82, 2799–2804. 10.1099/0022-1317-82-11-2799 11602792 PMC3795388

[ref74] Smith J. S. Lindsay L. Hoots B. Keys J. Franceschi S. Winer R.. (2007). Human papillomavirus type distribution in invasive cervical cancer and high-grade cervical lesions: a meta-analysis update. Int. J. Cancer 121, 621–632. 10.1002/ijc.22527, PMID: 17405118

[ref75] Varsani A. Williamson A. L. de Villiers D. Becker I. Christensen N. D. Rybicki E. P. (2003). Chimeric human papillomavirus type 16 (HPV-16) L1 particles presenting the common neutralizing epitope for the L2 minor capsid protein of HPV-6 and HPV-16. J. Virol. 77, 8386–8393. 10.1128/JVI.77.15.8386-8393.2003, PMID: 12857908 PMC165259

[ref76] Varsani A. Williamson A.-L. Jaffer M. A. Rybicki E. P. (2006a). A deletion and point mutation study of the human papillomavirus type 16 major capsid gene. Virus Res. 122, 154–163. 10.1016/j.virusres.2006.07.012 16938363

[ref77] Varsani A. Williamson A. L. Stewart D. Rybicki E. P. (2006b). Transient expression of human papillomavirus type 16 L1 protein in Nicotiana benthamiana using an infectious tobamovirus vector. Virus Res. 120, 91–96. 10.1016/j.virusres.2006.01.022 16530873

[ref78] Wang X. Wang Z. Christensen N. D. Dillner J. (2003). Mapping of human serum-reactive epitopes in virus-like particles of human papillomavirus types 16 and 11. Virology 311, 213–221. 10.1016/S0042-6822(03)00179-X, PMID: 12832218

[ref79] White W. I. Wilson S. D. Palmer-Hill F. J. Woods R. M. Ghim S.-j. Hewitt L. A.. (1999). Characterization of a major neutralizing epitope on human papillomavirus type 16 L1. J. Virol. 73, 4882–4889. PMID: 10233949 10.1128/jvi.73.6.4882-4889.1999 PMC112531

[ref80] World Health Organisation (2017). Cancer. Available at: http://www.who.int/cancer/en/ (Accessed April 4, 2017).

[ref81] Xia L. Xian Y. Wang D. Chen Y. Huang X. Bi X.. (2016). A human monoclonal antibody against HPV16 recognizes an immunodominant and neutralizing epitope partially overlapping with that of H16. V5. Sci. Rep. 6. 10.1038/srep19042, PMID: 26750243 PMC4707464

[ref82] zur Hausen H. (2002). Papillomaviruses and cancer: from basic studies to clinical application. Nat. Rev. Cancer 2, 342–350. 10.1038/nrc798, PMID: 12044010

