# Supplementary figures and images for: Substitution of Human Papillomavirus Type 16 L2 Neutralizing Epitopes Into L1 Surface Loops: The Effect on Virus-Like Particle Assembly and Immunogenicity
Source: Front Plant Sci. 2019 Jun 20;10:779. doi: 10.3389/fpls.2019.00779 (PMC6597877; doi:10.3389/fpls.2019.00779)

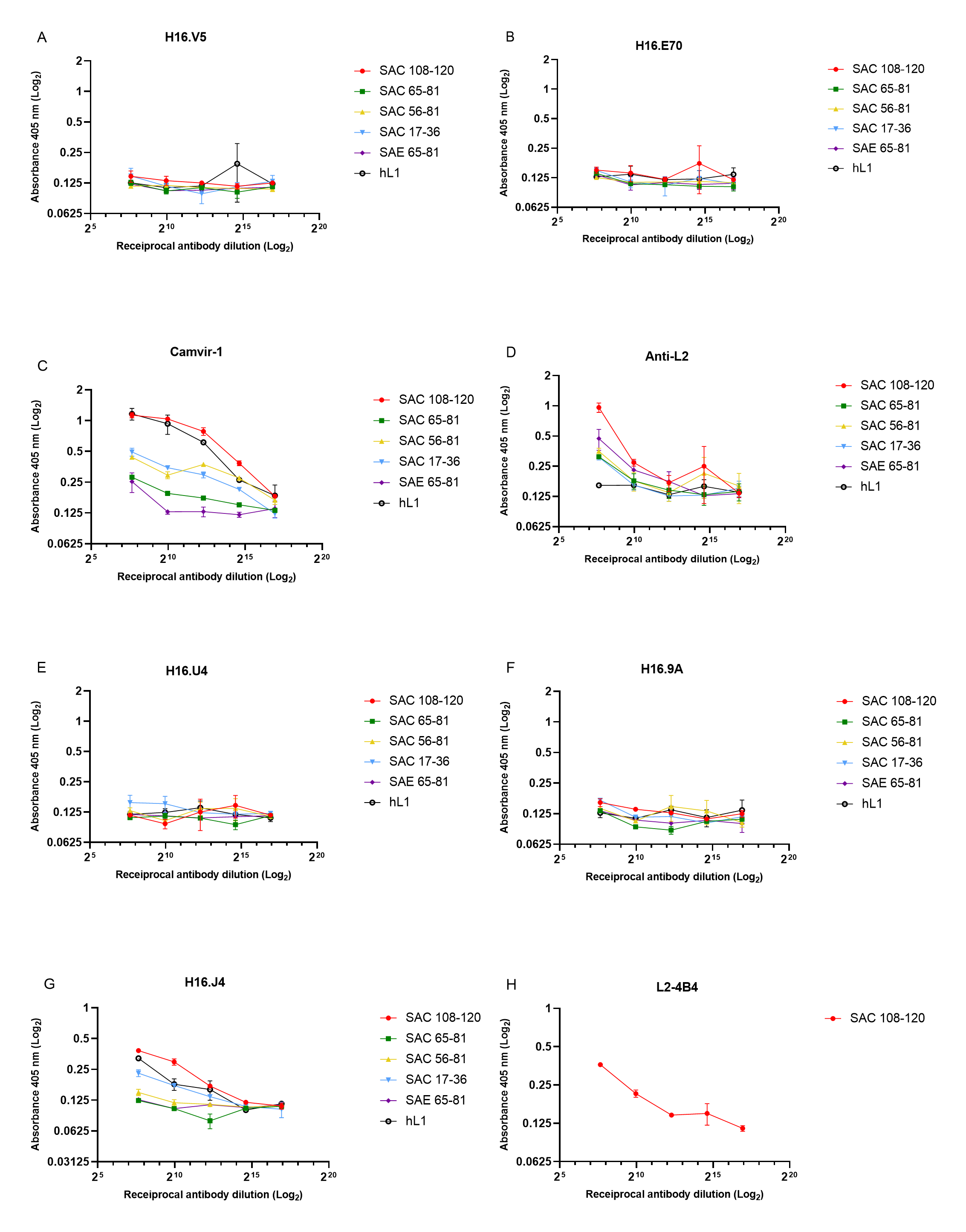

Supplement: Supplementary Figure 1 — Characterization of cVLP epitope display by indirect ELISA. Binding of monoclonal and polyclonal antibodies to HPV-16 L1:L2 cVLPs and HPV-16 L1 VLPs under denaturing conditions were analyzed in triplicate using conformational neutralizing mAbs H16.V5 (A), H16.E70 (B), H16.U4 (E), H16.9A (F), linear neutralizing mAb H16.J4 (G), non-neutralizing mAb Camvir-1 (C), mAb to L2 peptide 108-120 (L2-4B4) (H), and polyclonal anti-L2 serum (D). Error bars indicate standard deviation. [file Image_1.tif]

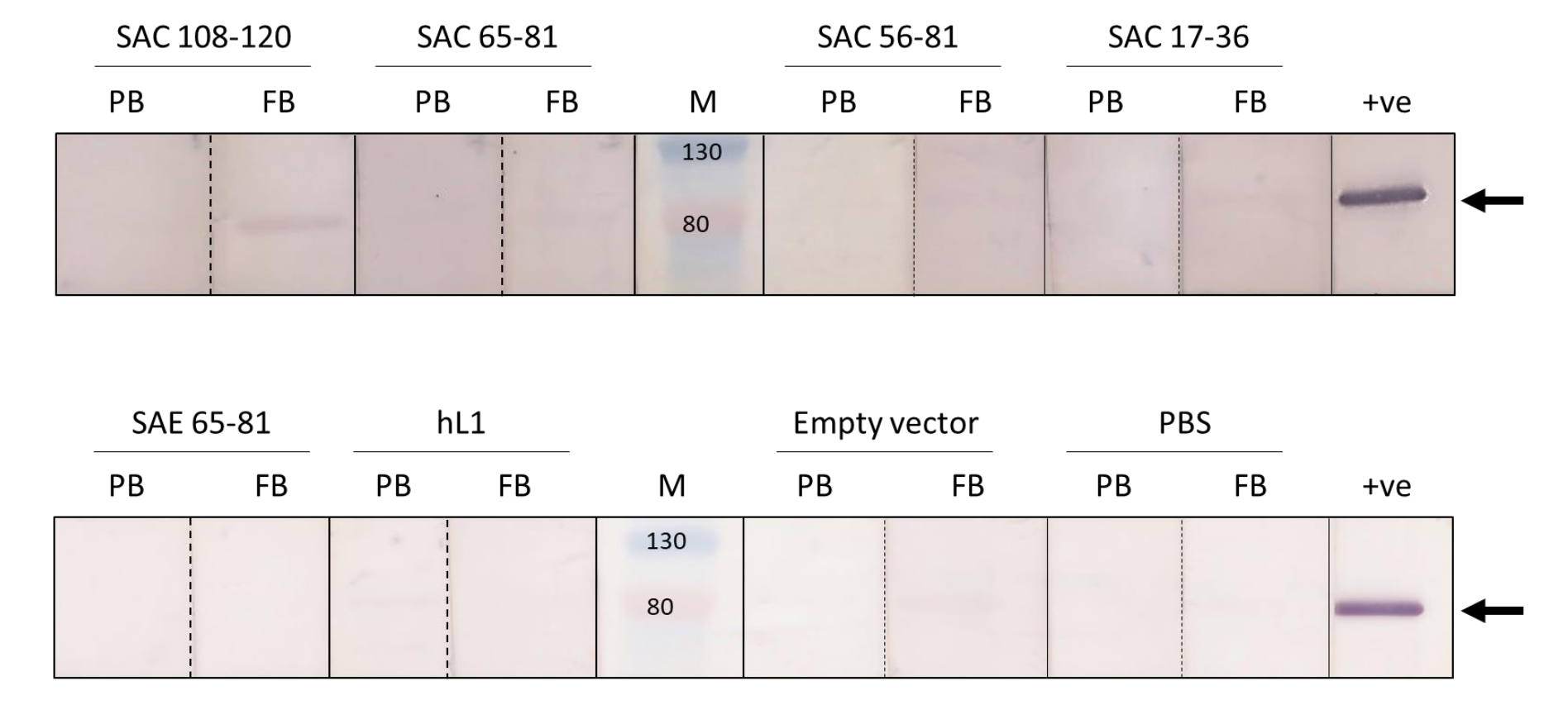

Supplement: Supplementary Figure 2 — Anti-L2 western blots using pooled mouse sera. HPV-16 L2 protein was probed with pre-bleed (PB) or final bleed (FB) sera at 1:2000 from the 8 vaccine groups. A band at 80 kDa is expected for L2 protein. Labels: M, molecular weight marker (kDa); +, L2 positive control detected with anti-His mAb (1:2000). [file Image_2.tiff]
